# Newborn Hearing Screening in Québec, Canada

**DOI:** 10.1044/2023_AJA-23-00041

**Published:** 2023-11-21

**Authors:** Gabriel Trottenberg, W. Robert J. Funnell, Hamid Motallebzadeh

**Affiliations:** a Faculty of Medicine and Health Sciences, McGill University, Montréal, QC, Canada; b Department of BioMedical Engineering, McGill University, Montréal, QC, Canada; c Department of Otolaryngology—Head & Neck Surgery, McGill University, Montréal, QC, Canada; d Department of Pediatric Surgery, McGill University, Montréal, QC, Canada; e Department of Communication Sciences & Disorders, California State University, Sacramento

## Abstract

**Purpose:**

This study discusses the history and current state of the newborn hearing screening program in Québec and aims to assess general challenges associated with establishing universal newborn hearing screening (UNHS) programs.

**Method:**

We reviewed the statistics of the occurrence and long-term effects of congenital hearing loss and the immediate and long-term benefits of UNHS and its limitations. The resources for this study included financial reports related to establishing UNHS in different health care systems; Canadian provincial, territorial, and federal regulations and publications; local and nationwide media; and interviews health care staff and program managers.

**Results:**

Because of its benefits and its cost-effectiveness, UNHS programs have been implemented in many health care systems around the world. Despite Canada's success in offering a wide array of health care services to its citizens, certain provinces trail behind others in developing UNHS programs. Although there have been recent improvements in the screening rate of the province of Québec, nearly half of all Québec newborns continue to not be screened for hearing loss. The reasons for the current low screening rate include delays in implementation, information-technology complications, operating costs, and lack of public awareness.

**Conclusions:**

For UNHS to be implemented in a timely fashion, those involved in the process should first understand what challenges may arise. Québec's experience with this process may provide useful lessons for other health care systems.

Approximately one to three per 1,000 newborns are born with permanent congenital hearing loss (e.g., Patel et al., 2011). A lack of auditory stimulation early in life causes the brain to reorganize, often resulting in poor long-term language and communication outcomes among children with delayed diagnoses of hearing loss. These individuals are consequently at increased risk of experiencing various neurodevelopmental issues, involving not only language and communication but also sequential memory, abstract reasoning, and even executive functioning (Bower et al., 2023). Ultimately, this may result in several lifelong complications for these individuals, including poor academic performance, difficulties in establishing healthy relationships, and various other socioemotional challenges. As such, detection of hearing loss at an early age, followed quickly by appropriate treatment, can mitigate these negative impacts (e.g., Wake et al., 2016).

Early hearing detection and intervention (EHDI) programs are designed to reduce the age at which hearing loss is diagnosed and to provide infants affected by hearing loss with timely access to appropriate interventions. Since roughly half of newborns with hearing loss do not present with risk factors for this impairment at the time of their diagnosis (Joint Committee on Infant Hearing [JCIH], 2019; Ministère de la Santé et des Services sociaux [MSSS], 2019a), universal newborn hearing screening (UNHS) is an important first step for any EHDI program. The JCIH (Declau et al., 2008) recommends that all EHDI programs follow at least a “1–3–6” guideline (meaning that all newborns should undergo hearing screening by 1 month of age, receive an audiological diagnosis by 3 months of age, and be treated for hearing loss by 6 months of age). Furthermore, programs should strive to meet a “1–2–3” target once they consistently achieve the “1–3–6” guideline. Later surveillance is also important to identify hearing loss, which may have been missed in the newborn screening or which has a delayed onset (e.g., Bielecki et al., 2011; MSSS, 2019b). An example of later surveillance is screening of speech and language developmental milestones during routine pediatric visits (JCIH, 2019).

UNHS has been shown to reduce the median age of a newborn's diagnosis of hearing loss from 24 months to 3 months and to lower the median age at which interventions for hearing loss begin from 24 months to 6 months (Patel et al., 2011). This can have significant long-term positive impacts on individuals with hearing loss. For instance, Yoshinaga-Itano et al. (2017) used the vocabulary quotient (VQ; vocabulary age divided by chronological age times 100) to compare two groups of infants with hearing impairments. The first group was screened and treated according to appropriate EHDI guidelines, while the second group was not. The first group had a mean VQ of 79.2 compared to only 67.9 for the second group.

Despite longstanding efforts that began over a decade ago to improve access to hearing screening throughout its health care system, the province of Québec in Canada still only screens about half of its newborns. This clinical focus article presents a brief overview of UNHS, the UNHS situation in Canada, and then the historical context and current situation of UNHS in Québec, with the goal of identifying challenges that may also be faced by other jurisdictions in the process of implementing UNHS. The resources for this study included financial reports related to establishing UNHS in different health care systems; Canadian provincial, territorial, and federal regulations and publications; local and nationwide media; and interviews with health care staff and program managers.

## Overview of UNHS

Newborn hearing screening is typically based on the use of one or both of two distinct methods: otoacoustic emission (OAE) testing and auditory brainstem response (ABR) testing (JCIH, 2019). OAE tests use a microphone in the external ear canal to measure the acoustic responses produced by the outer hair cells in the cochlea and assess the integrity of the three peripheral parts of the auditory system (outer, middle, and inner ear). ABR testing not only evaluates the same peripheral parts of the ear as OAE but can also assess the integrity of the neural pathways from the cochlea to the auditory brainstem. The test involves the use of electrodes placed on the infant's scalp to detect whether neurological activity is present in response to sounds. Failure of either OAE or ABR indicates potential conductive and/or sensorineural hearing loss. Both OAE and ABR tests are automated when used for screening and provide pass or fail results.

Screening tests sometimes lead to results that are false positives, in the sense that the newborn does not have a permanent hearing loss that requires treatment. For instance, the presence of amniotic fluid in the middle ear following birth can block the conduction of the test stimuli to the cochlea. Such results create additional health system costs and may cause unnecessary parental anxiety. Good screening programs can attain referral rates as low as around 2% (e.g., Akinpelu et al., 2014) but, given a congenital hearing loss incidence of, say, two per 1,000, even a referral rate of 2% implies that about 10 newborns would be referred for an audiological follow-up for every actual case of hearing loss requiring treatment. Thus, various guidelines and techniques have been developed over the years to minimize the false-positive rates generated by hearing screening tests. For example, screening a newborn 48 hr or more after birth gives a higher specificity than when the screening is performed less than 24 hr after birth, since this allows more time for amniotic fluid to clear from the infant's middle ear (Van Dyk et al., 2015). This is especially true of OAE testing, because both stimuli and responses must traverse the middle ear (Motallebzadeh & Puria, 2022). However, such later screening is often not possible, since many infants are discharged from the hospital before the 48-hr mark. Combining both OAE tests (which are faster and less expensive) and ABR tests (which are less susceptible to false positives) has been shown to improve the specificity of hearing screening programs while maintaining cost-efficiency. For example, Lin et al. (2005) showed that a procedure involving both a transient evoked OAE (TEOAE) component and an ABR component reduced the referral rate to 1.8% compared to 5.8% for a two-step TEOAE procedure.

Certain benefits of UNHS can be difficult to monetize. Examples of such benefits include improvements in social skills, increased success in education and in professional careers, and greater feelings of fulfillment and satisfaction in life. A number of studies have nonetheless attempted to estimate whether UNHS is cost-effective in comparison to risk factor–based screening programs. For example, Yoshinaga-Itano et al. (2021) reviewed four articles that analyzed the cost-effectiveness of UNHS programs: (a) Mehl and Thomson (2002) calculated that the screening costs of UNHS in the United States can be recovered by the 10th year following the program's implementation and would subsequently result in societal savings, including a 50% reduction in educational costs; (b) Keren et al. (2002) estimated that improving language outcomes through early intervention could result in a reduction of the lifetime cost of deafness of an individual in the United States by approximately $430,000 (USD); (c) Schroeder et al. (2006) measured the economic costs of hearing loss among children in the United Kingdom between the ages of 7 and 9 years and found that UNHS resulted in a mean societal saving of £2,213 (GBP) per person per year; and (d) in a study on the costs of hearing loss in teenagers in Southern England, Chorozoglou et al. (2018) calculated a mean cost reduction of £3,594 (GBP) per person per year with the use of UNHS. Thus, all four studies came to the overall conclusion that the implementation of UNHS can represent a cost-saving advantage to society. Most recently, in their “World Report on Hearing,” the World Health Organization (2021) “conservatively estimated” that UNHS results in a return on investment of 1.67 dollar/dollar for a lower middle-income country and 6.53 for a high-income country.

Overall, despite the consequences associated with false-positive rates, the benefits of UNHS are widely considered to outweigh its costs. In view of the efficiency, practicality and positive societal impacts of UNHS, many health care systems around the world have put such programs into practice. According to a survey by Neumann et al. (2020), newborn hearing screening rates were high in many countries in 2020 and continued to follow an upward trend; among the G7 nations, France, Germany, the United Kingdom, and the United States had achieved screening rates of 95% or greater, while Japan (62%), Canada (64%), and Italy (80%) had not.

## UNHS in Canada

Each Canadian province and territory has jurisdiction over its own health care system, and some of them trail behind others in implementing UNHS programs. In a recent “report card” prepared by the Canadian Infant Hearing Task Force (CIHTF, 2019), which summarizes the current state of EHDI throughout the country, a “Sufficient” EHDI program was defined as one that (a) provides hearing screening to all newborns, (b) successfully detects infants with hearing loss, (c) provides timely intervention services as well as support for families, and (d) continuously monitors its own progress. In this report card, the country as a whole was deemed “Insufficient” in providing its population with adequate EHDI services; according to the report card, only six of the 13 provinces and territories had implemented “programs that were ‘Sufficient’” in the sense of having fulfilled all four criteria.

Nonetheless, most provinces and territories have succeeded in establishing high screening rates for hearing loss: the Task Force recorded rates in 2019 of approximately 90% or more in Alberta, British Columbia, Manitoba, New Brunswick, the Northwest Territories, Nova Scotia, Ontario, Prince Edward Island, and Yukon. Québec is Canada's second largest province, with a population of about 8.6 million, but, as of July 24, 2023, only 62% of newborns in Québec were being screened for hearing loss (Direction Santé Mère Enfant, MSSS). These numbers fall well short of the rates recorded in the largest and third largest provinces, Ontario and British Columbia, which screened 94% and 97% of their newborns, respectively, according to CIHTF (2019). For both provinces, comprehensive protocols are available online for both screening (Bagatto et al., 2019; Hyde et al., 2019) and audiological follow-up (Bagatto et al., 2020; Hatton et al., 2022).

## History of Newborn Hearing Screening in Québec

The history of newborn hearing screening in Québec dates back to 2008, when the Centre hospitalier (CH) universitaire Sainte-Justine (CHUSJ) and the McGill University Health Centre announced that they would both begin screening newborns for hearing loss in the absence of a province-wide UNHS program (CIHTF, 2019). Around the same time, the Québec government began evaluating whether the establishment of a province-wide UNHS program would be feasible. In a report submitted to the MSSS, the Institut national de santé publique du Québec (INSPQ, 2008) estimated the benefits and costs of developing a UNHS program for Québec and used a study on newborn hearing screening in England (Bamford et al., 2004) as a model for their calculations. The INSPQ estimated that around 84 babies are born annually in Québec with hearing loss and that a well-developed UNHS program would be able to detect 72 of these cases. The ability to detect only 72 of the 84 newborns would be explained by a screening rate of less than 100%, some false-negative results, and potential losses to follow up. This detection rate was considered to be a substantial improvement over the then-current screening situation that, according to the INSPQ, was leading to only 28 of the 84 cases being detected. The report also estimated that roughly 90% of the 72 newborns identified as having hearing loss would be able to receive appropriate treatment for their condition before the age of 7 and a half months.

The INSPQ also estimated how a UNHS program in Québec would financially impact the province. By subtracting the cost of implementing a UNHS program from its overall monetary and nonmonetary benefits, the INSPQ concluded that having a UNHS program in Québec would create a net societal surplus of more than $1,700,000 (CAD) per year. The INSPQ concluded that the benefits of UNHS outweighed its potential drawbacks and recommended that MSSS implement a UNHS program throughout Québec.

Since the 2008 INSPQ report, Québec has developed an EHDI program known as the Programme québécois de dépistage de la surdité chez les nouveau-nés (PQDSN). The development of the PQDSN started with a pilot program. This phase, which began in 2013, involved the participation of four hospital centers (CHUSJ, CH Nord de Lanaudière, CH Hôtel-Dieu de Sorel, and CH Pierre Boucher). These institutions were selected because they were all in the same integrated university health network (Réseau universitaire intégré de santé), had compatible patient admission-discharge-transfer (ADT) information systems, and ranged from more than 4,000 births per year, including Levels 3 and 4 neonatal intensive care units (NICU's), to less than 500 births per year, including a Level 1a NICU.

Following the pilot phase, which was scheduled to last for 1 year, the goal of the MSSS was to implement the PQDSN throughout the entire province in a timely fashion. However, due to various issues that are discussed below, by July 24, 2023, only 20 of the 81 birthing institutions in Québec were offering the PQDSN to their newborns, including five institutions having ≤ 1,000 births/year and seven having ≥ 3,000 births/year (Direction Santé Mère Enfant, MSSS). Many institutions throughout the province still do not offer UNHS.

## PQDSN Screening Procedure

The PQDSN's reference document, the “Cadre de référence” (MSSS, 2019a) specifies two different protocols, depending on whether or not the newborn being screened has any risk factors for hearing loss. The risk factors that are listed in the “Cadre de référence” include, but are not limited to, a positive family history for hearing loss, congenital cytomegalovirus, extended stays in a NICU, and prematurity (MSSS, 2019a, app. 2).

In the case where a newborn does not possess risk factors and can be screened during their initial stay in the hospital, the PQDSN's screening procedure begins with an initial automated distortion-product OAE (ADPOAE) test, which ideally is performed at least 24 hr after birth. As previously described, allowing more time between the birth of the newborn and the time of the test allows more time for amniotic fluid to clear from the infant's ear. If the ADPOAE test leads to a fail result in either one or both ears, a second identical ADPOAE test is performed, ideally at least 4 hr after the first one. If this second test gives another fail result in one or both ears, an automated ABR (AABR) test is performed. A fail result in this AABR test leads to the outpatient stage of the protocol, which involves an AABR test performed 2 weeks following the date of the most recent inpatient screening. If the outpatient test is failed, the newborn is referred for a comprehensive audiological evaluation. As previously discussed, UNHS procedures that combine the use of both OAE and ABR testing have been shown to be effective in minimizing false positive rates.

The process of screening newborns who have at least one risk factor for hearing loss is different from the protocol described above. It involves screening only with AABR or, in cases of certain risk factors such as meningitis, bypassing the screening altogether and proceeding directly to an audiological assessment (MSSS, 2019a, p. 12). Many UNHS programs currently use similar protocols for newborns with risk factors (Bagatto et al., 2019).

Finally, if a newborn cannot be screened during their initial stay at the hospital, they are provided with a follow-up appointment for their screening as soon as possible. In this outpatient scenario, the protocol is applied in the same way as it would have been for an inpatient case, except that the newborn does not receive a second ADPOAE test or a second AABR test. The outpatient screening is performed by institutions offering the PQDSN and, thus, total screening rates recorded by the PQDSN's database include all newborns having undergone testing, whether inpatient or outpatient. Unfortunately, a child born in an institution that has not yet implemented the PQDSN is unable to obtain such a screening appointment.

According to the “Cadre de référence” (MSSS, 2019a), institutions having implemented this program should aim to screen at least 95% of their newborns before the age of 1 month and to achieve a referral rate of less than 2%. The CHUSJ, for example, has indeed met both of these targets.

With an overall provincial screening rate of 62% as mentioned above, the challenges facing the PQDSN seemingly do not lie with the program's screening procedures, but rather with its accessibility. Based on interviews that we conducted with key figures involved in the development and current management of the PQDSN as well as on financial reports (INSPQ, 2008), we have identified four main reasons that may have contributed to the program's low screening rate.

## Reasons for Continued Low Screening Rates in Québec

### A. Delays in Implementation

Although Phase 1 of the implementation of PQDSN was supposed to be completed in 2014, complications surrounding the creation and unification of PQDSN databases (explained in more detail in Section B) delayed the completion of Phase 1 until 2017. Once the pilot phase had been completed, the PQDSN began progressing more rapidly, as more birthing centers began implementing the program. However, the COVID-19 pandemic put a sudden halt to the PQDSN's expansion, with health care institutions and officials of the MSSS needing to invest their time, attention, and resources into other issues. The pandemic situation has now improved and progress is accelerating.

### B. Information Technology Complications

If an institution decides to implement the PQDSN, the government covers the costs of the screening tools and human resources necessary for the program. However, each institution is responsible for integrating their ADT system with the PQDSN's database and must pay for any associated information technology project. Many different ADT systems are used in Québec's health care institutions, including some that were developed within the institutions themselves, and each must be adapted to the PQDSN. This renders the unification of the province-wide program a logistical challenge.

### C. Operating Costs of UNHS

As previously indicated, the INSPQ argued in 2008 that the implementation of UNHS in Québec could make a substantial long-term improvement to the province's health care budget (INSPQ, 2008). However, there continues to exist a shortage of health care staff, including the nurses who would normally perform the screening. As an alternative to simply hiring more nurses, it might be possible to find other personnel to do the screening, such as nursing assistants or technicians, but they also are in short supply. Either approach would put additional short-term pressure on the health care system's already strained budget.

### D. Lack of Awareness

Many current and future parents and even some pediatricians appear to be unaware of UNHS and its benefits to newborns. Although certain organizations in the province, such as the Association du Québec pour enfants avec problèmes auditifs (AQEPA) and the Ordre des orthophonistes et audiologistes du Québec, and also media outlets (e.g., Blias, 2021; Mercier, 2022), have attempted to raise awareness, UNHS seemingly remains relatively unknown among Québec's general population. In turn, the government seemingly does not receive much pressure from the public to consider the urgency of this situation, which could have contributed to the province's slow progress in developing the program.

## Conclusions

With each passing year, more and more Québec-born children with hearing loss have not received the necessary screening tests to identify their condition at an appropriate age. These children are often left to face a life filled with communicative and socioemotional challenges. Stories of Québec children who have suffered the consequences of delayed diagnoses of hearing loss have been reported in the past (e.g., Blias, 2021; Cousineau, 2021), but Québec continues to lag while other jurisdictions have had tremendous success in providing their newborns with universal hearing screening in the last few decades.

On May 11, 2021, the Québec National Assembly passed a motion that promised that UNHS would be available to all newborns throughout the province by the end of 2021 (AQEPA, 2017). Although this was a promising step in the right direction, the target date has passed and almost half of Québec newborns remain unscreened for hearing loss. The COVID-19 pandemic obviously drained a large portion of the resources of the health care system in Québec, as it did all around the world, and this no doubt contributed to the failure to attain the motion's objective. The pandemic has also highlighted systemic limitations within Québec's health care system, and there is a growing desire among the general population to improve it; hearing screening for all Québec newborns would be an important part of such improvements. In the Québec government's budget presented in March 2022, health care was indeed the largest single component of additional spending, including significant investments in information technology (Finances Québec, 2022).

The benefits of screening newborns for hearing loss at a young age cannot be overstated, and implementing UNHS should be a priority for all health care regions that have yet to do so. However, for UNHS to be implemented in a timely fashion, those involved in the process should first have an understanding of what challenges may arise. This review of the history and current status of hearing screening in Québec highlights some of these challenges and may provide useful lessons for other health care systems.

## Data Availability Statement

The data that support the findings of this study are publicly available financial reports; Canadian provincial, territorial, and federal regulations and publications; and local and nationwide media provided in the reference list. The interviews with health care staff were online.
